# The Medical, Surgical, and Hygienic Exhibition, 1901

**Published:** 1901-06-08

**Authors:** 


### THE INSTITUTIONAL WORKSHOP.

### THE MEDICAL, SURGICAL, AND HYGIENIC EXHIBITION, 1901.

THIS annual exhibition, which was the seventh of its kind, was held on the four days from Tuesday 28th to Friday 31st May, and was in every way more successful than those of previous years. The exhibits comprehended a wide range, and were more in keeping with the title of the exhibition than in the past, while the increased attendance of medical men and nurses must have given much satisfaction and encouragement to the promoters.

There was an extensive and well-arranged show of drugs and medical preparations, surgical instruments and appliances, antiseptic and other surgical dressings and bandages; a variety of milk, meat, diabetic and other foods and essences; an abundance of medical, scientific, and nursing literature, and some very interesting and ingenious sanitary appliances and specialities.

It may be mentioned, in passing, that a change in the constitution of this exhibition is contemplated, which, if carried into effect, will establish it as a permanent undertaking, capable of such development as may be required by the advance of science, invention, and commercial enterprise, and the growth in professional interest. The strides which have been made, year by year, give indications of a large extension and increased popularity, and it therefore is well that the authorities should anticipate the responsibilities which would be entailed by further expansion.

Commencing with drugs, Messrs. Oppenheimer, Son & Co., Limited, 179 Queen Victoria Street, E.C., gave an interesting display, including their well-known palatinoids and bipalatinoids, which are the specialities of this firm, but their most important exhibit was Traumatol (cresylic oxide), which is a powerful germicide, free from objectionable odour, and designed to supersede iodoform.

Messrs. Parke, Davis & Co., 111 Queen Victoria Street, E.C., gave an exhibit of their chemical compounds, standardised fluid extracts, and euthymol toilet preparations. Attention was directed to their antidiphtheria serum, which is certified in the laboratories of the Royal College of Physicians (Lond.) and supplied in hermetically-sealed bulbs.

Messrs. Fairchild, Bros. & Foster, Snow Hill Buildings, E.C., had on view their digestive products for which they are justly renowned. The newest preparation was Diazyme, an animal diastasic essence; but the most important item was their Panopepton, which for its restorative and stimulating properties has been found so valuable.

Messrs. Arthur & Co., 69 Berners Street, W., gave prominence to their Bromaurum and Hydraurum preparations, for employment in the treatment of rheumatism and other affections.

Mr. E. Merck, of Darmstadt had a show case containing specimens of their chemicals and drugs which are generally known to be of a high order.

The Abbey Effervescent Salt Company, Limited, 144 Queen Victoria Street, E.C., gave a considerable display of their saline aperient, which appears to be advancing in public favour.

Other exhibitors in this class included Messrs. M. & L. Hoff, of Hamburg, and 29 New Bridge Street, E.C., with malt extracts, and Dr. Enoch's Liqueur Ironol (which is a palatable tonic), and Edgar's Lotion, Limited, Dartford, who had two well-arranged stalls containing their lotion, which attracted a large number of visitors.

Messrs. Cooper & Co., 80 Gloucester Road, South Kensington, S.W., gave a most attractive show of their "Globenaris" oxy-carbonated mineral waters, "Globena" meat essences, &c. The mineral waters, which claim special mention for their tested purity and high quality, are supplied in syphons, the taps of which are lined with porcelain.

Messrs. Down Brothers, St. Thomas's Street, S.E., exhibited a very fine series of surgical instruments, including many ingenious inventions of recent production. This firm gained the highest award for their exhibit at the Paris Exhibition, 1900.

The stand of Mr. Frank A. Rogers, 327 Oxford Street, W., was, for the most part, devoted to sprays of various kinds, but particular attention was invited to his "Hypodermules," small hermetically-sealed glass bulbs containing a measured dose of sterilised solution for hypodermic injection.

Mr. W. K. Stacy, 19 Newgate Street, E.C., instrument maker, gave an extensive display of his manufactures, which occupied two stalls. The "Queen's" nurses' bag attracted marked attention from doctors as well as nurses. This bag has interchangeable linings, and the fittings, which are well suited to emergencies, are arranged with the greatest economy of space. There was also a very fine show of nurses' wallets, of aluminium and of leather.

Scientific instruments were exhibited by Harry W. Cox, Limited, of Cursitor Street, Chancery Lane, W.C., and A. Rosenberg & Co., 21 Southampton Row, Holborn, who gave demonstrations with their X-ray apparatus.

Much attention was devoted to the exhibit of the Dowsing Radiant Heat Company, 24 Budge Row, E.C., who had on view a fully-equipped bed with the radiant heat in operation, and other appliances, illustrating their system of treatment which is in operation in various parts of the country.

Messrs. Anderson, Anderson, and Anderson, Limited, 37 Queen Victoria Street, E.C., exhibited a selection of indiarubber and waterproof goods, which were chiefly adaptable to the requirements of hospitals, asylums, and other institutions. Their new waterproof sheet, with central tube for draining purposes, claims special mention, as also does the doctor's operation apron, which fastens with elastic cross straps instead of tapes.

Mr. Vincent Wood, 4 Albion Place, Blackfriars Bridge, S.E., had a stall containing surgical bandages, appliances and belts, elastic hosiery, &c. Attention was drawn to his "Eureka" crêpe Velpeau bandage, which maintains equal pressure wherever applied, and has the advantage of being porous and washable.

The Sanitary Wood Wool Company, Limited, 26 Thavies Inn, E.C., exhibited their widely-known manufactures of Hartmann's wood wool preparations, including antiseptic towelettes, tissue, wadding, &c., and the complete sanitary outfit for accouchement. This stall attracted the notice of a large number of nurses and medical men.

The Domen Belt Company, 456 Strand, W.C., exhibited a variety of abdominal belts and corsets, prominent among which were the "Domen" straight-fronted corset with belt attachment, which is specially applicable in cases of gastric trouble and corpulency.

The Portia Company had on view their combined stocking, suspender and shoulder support, which is easily adjustable to any figure.

Mr. Thomas Holland, 40 South Audley Street, W., was showing his improved instep arch sock—an elastic appliance for use in the case of flat foot—and boots made in the natural shape of the feet.

The Cellular Clothing Company, Limited, 72 Fore Street, E.C., showed specimens of "Aertex" cellular clothing, including underwear, corsets, and sheeting. Many advantages are claimed for this clothing, which is light, comfortable, and healthy, owing to the presence of air in its meshes.

(*To be continued.*)

